# Supplementary material for: Accurate Measurement of Copper Overload in an Experimental Model of Wilson Disease by Laser Ablation Inductively Coupled Plasma Mass Spectrometry
Source: Biomedicines. 2020 Sep 16;8(9):356. doi: 10.3390/biomedicines8090356 (PMC7555421; doi:10.3390/biomedicines8090356)
Supplement: Supplementary file 1 [file biomedicines-08-00356-s001.zip › Table S1.docx]

**Table S1: Element concentrations as determined by LA-ICP-MS**

| **Genotype** | **Age**  **(weeks)** | **Animals**  **measured** | **Element (µg/g) Mean ± SD** | | | | | | | | | | |
| --- | --- | --- | --- | --- | --- | --- | --- | --- | --- | --- | --- | --- | --- |
|  |  |  | **^23^Na** | **^24^Mg** | **^31^P** | **^34^S** | **^39^K** | **^44^Ca** | **^52^Cr** | **^55^Mn** | **^56^Fe** | **^63^Cu** | **^64^Zn** |
| Wild type | 9 | 3 | 1329  ±  259.97 | 499.67  ±  48.60 | 11747  ±  1226.22 | 2760.67  ±  229.69 | 4231.67  ±  364.70 | 23.33  ±  1.62 | 0.58  ±  0.05 | 1.07  ±  0.14 | 549.33  ±  57.11 | 3.41  ±  0.28 | 37.33  ±  2.88 |
| Wild type | 13 | 3 | 1440.67  ±  172.22 | 559  ±  23.85 | 13133  ±  572.46 | 3044  ±  117.68 | 4660.67  ±  248.66 | 25.1  ±  1.02 | 0.60  ±  0.02 | 1.19  ±  0.05 | 449  ±  78.80 | 3.45  ±  0.30 | 39.7  ±  1.84 |
| Wild type | 36 | 5 | 762  ±  167.96 | 267.2  ±  38.74 | 5562.4  ±  738.97 | 1200.8  ±  165.57 | 2180.4  ±  268.75 | 27.31  ±  22.29 | 0.35  ±  0.02 | 0.58  ±  0.07 | 371  ±  96.44 | 2.09  ±  0.28 | 22.15  ±  3.86 |
| *Atp7b^-/-^* | 20 | 5 | 1344.2  ±  113.58 | 246.8  ±  15.29 | 5020  ±  251.55 | 1141.8  ±  38.32 | 2091  ±  81.37 | 13.13  ±  0.95 | 0.06  ±  0.004 | 0.69  ±  0.10 | 314.2  ±  37.88 | 112.72  ±  13.26 | 43.16  ±  3.47 |
